# Assessing the causal relationships between gout and hypertension: a bidirectional Mendelian randomisation study with coarsened exposures

**DOI:** 10.1186/s13075-022-02933-4

**Published:** 2022-10-29

**Authors:** Benjamin Lai, Huang-Ping Yu, Yu-Jing Chang, Liang-Chin Wang, Che-Kai Chen, Weiya Zhang, Michael Doherty, Shang-Hung Chang, Jun-Te Hsu, Kuang-Hui Yu, Chang-Fu Kuo

**Affiliations:** 1 Center for Artificial Intelligence in Medicine, Chang Gung Memorial Hospital, Taoyuan, Taiwan; 2 Department of Anesthesiology, Chang Gung Memorial Hospital, Taoyuan, Taiwan; 3 College of Medicine, Chang Gung University, Taoyuan, Taiwan; 4 Division of Rheumatology, Allergy and Immunology, Chang Gung Memorial Hospital, Taoyuan, Taiwan; 5 Academic Rheumatology, School of Medicine, University of Nottingham, Nottingham, UK; 6 Pain Centre Versus Arthritis, University of Nottingham, Nottingham, UK; 7 Division of Cardiology, Chang Gung Memorial Hospital, Linkou Medical Center, Taoyuan City, Taiwan; 8 Center for Big Data Analytics and Statistics, Linkou Medical Center, Chang Gung Memorial Hospital, Taoyuan City, Taiwan; 9 Graduate Institute of Nursing, Chang Gung University of Science and Technology, Taoyuan City, Taiwan; 10 Department of General Surgery, Gung Memorial Hospital at Linkou, Chang Gung University College of Medicine, Taoyuan 333, Chang, Taiwan

**Keywords:** Gout, Hypertension, Mendelian randomisation

## Abstract

**Objectives:**

Observational studies have demonstrated associations between gout and hypertension, but whether they are causal remains unclear. Our work aims to assess the causal relationship between gout and hypertension.

**Methods:**

We obtained genetic information from the Taiwan Biobank, including 88,347 participants and 686,439 single-nucleotide polymorphisms (SNPs). A novel model of Mendelian randomisation (MR) with coarsened exposures was used to examine the causality between the liability of gout on hypertension and vice versa, using 4 SNPs associated with gout and 10 SNPs associated with hypertension after removal of SNPs associated with measured confounders. The binary exposure (gout/hypertension) can be considered a coarsened approximation of a latent continuous trait. The inverse-variance weighted (IVW) and polygenic risk score (PRS) methods were used to estimate effect size. The MR analysis with coarsened exposures was performed with and without adjustments for covariates.

**Results:**

Of the 88,347 participants, 3253 (3.68%) had gout and 11,948 (13.52%) had hypertension (men, 31.9%; mean age 51.1 [SD, 11.1] years). After adjusting to measured confounders, MR analysis with coarsened exposures showed a significant positive causal effect of the liability of gout on hypertension in both the IVW method (relative risk [RR], 1.10; 95% confidence interval [CI], 1.03–1.19; *p* = 0.0077) and the PRS method (RR, 1.10; 95% CI, 1.02–1.19; *p* = 0.0092). The result of causality was the same before and after involving measured confounders. However, there was no causal effect of the liability of hypertension on gout.

**Conclusions:**

In this study, we showed that the liability of gout has a causal effect on hypertension, but the liability of hypertension does not have a causal effect on gout. Adequate management of gout may reduce the risk of developing hypertension.

**Supplementary Information:**

The online version contains supplementary material available at 10.1186/s13075-022-02933-4.

## Introduction

Gout is a common inflammatory arthritis caused by the deposition of monosodium urate around the joints due to persistent elevation of serum urate (uric acid) levels above the saturation point for crystal formation. Gout has an incidence of 2.74 per 1000 person-years and is linked to increased risks of mortality and multiple comorbidities [1]. Hypertension and cardiovascular diseases are among the most common comorbidities of gout and hyperuricaemia [2, 3]. Multiple studies have attempted to explain the association by showing that gout or hyperuricaemia is associated with arterial stiffness [4], insulin levels [5], alterations in the renin-angiotensin system [6], and endothelial dysfunction [7]. Conversely, patients with hypertension have an increased risk of gout [8], implying a possible bidirectional causal relationship between gout and hypertension. However, because most of these studies were observational, it is difficult to ascertain the causality between gout and hypertension. Clinical trials investigating the effects of urate-lowering therapies (e.g., allopurinol and febuxostat) on blood pressure have reported conflicting results [9, 10].

Randomised controlled trials are the “gold standard” for inferring causal effects in medical research because random group assignments can minimise confounding bias; however, this may be expensive and not easy to implement. Mendelian randomisation (MR) mimics the random group assignment process by using randomly distributed single-nucleotide polymorphisms (SNPs) in genetic data as instrumental variables (IV) for group assignment [11–13]. Because most genetic SNPs are distributed randomly at birth and precede the outcomes of interest, exposure based on SNPs can minimise confounding biases and help to establish causality. The MR approach has been used to investigate the causal effects of multiple risk factors in a range of diseases [14, 15]. However, the causality between gout and hypertension has not been studied. A recent phenome-wide MR study demonstrated strong pairwise associations between urate and multiple cardiometabolic diseases [16], but causality between urate levels and hypertension was not established [16]. Another MR study suggested body mass index (BMI) has a causal effect on urate and hyperuricaemia and may confound urate and the development of urate conditions [17]. Other MR studies detected possible bias due to pleiotropic effects or intermediate factors [16, 18, 19].

Notably, the MR study design is used primarily for continuous exposures such as urate level and BMI, and its application to dichotomous variables may lead to erroneous measurement of effect size [20]. Therefore, we implemented a modified MR method with coarsened exposures based on the Falconer framework [20], which allowed us to use categorical variables as exposures. The method was used to investigate the bidirectional causal effects between gout and hypertension using the Taiwan Biobank, a national database containing the genetic information of Taiwanese individuals, together with multiple MR specifications to strengthen the results and conclusions.

## Materials and methods

### Data source

The study was based on genetic and clinical data from the Taiwan Biobank, which was established in 2012. The participants are adult Taiwan residents greater than 20 years of age. The Taiwan Biobank prospectively enrolls individuals from communities across Taiwan, with 142,882 participants as of 28 February 2021. All participants attended 1 of 39 enrollment centers; at these centers, each participant completed a series of genetic, physical, sociodemographic, and medical assessments. A standardised questionnaire was completed by each participant to record past medical history, medication, family history, and home environmental factors. Additionally, the Taiwan Biobank includes a physical examination and laboratory tests, including blood and urine tests performed at enrollment and follow-up visits, which provide sufficient variables for covariate examination. Another benefit of the Taiwan Biobank is its relatively homogenous population group compared to other large biobanks [21]. The prevalence of gout in Oceanian countries and Taiwanese aboriginals is also higher than those in North America and European countries [22].

### Genotyping and phenotyping

Genotyping was based on the C2-58 Axiom Genome-Wide TWB 2.0 Array containing 686,439 SNPs, specifically designed for people of Taiwanese descent (Thermo Fisher Scientific, Waltham, MA, USA). The data were anonymized, and no tracking was possible. Information regarding each participant’s personal and clinical history was collected by questionnaires at enrollment, then every 2 years thereafter. We obtained data concerning 88,347 participants. We also obtained various data associated with gout and hypertension, including each participant’s age, sex, BMI, and serum creatinine levels. In Taiwan, the diagnosis of gout is based on clinical observation of rapidly developing monoarticular arthritis, and the Taiwan Biobank then used questionnaires to ask whether the participants have a history of gouty arthritis.

### SNP selection

**Fig. 1:**
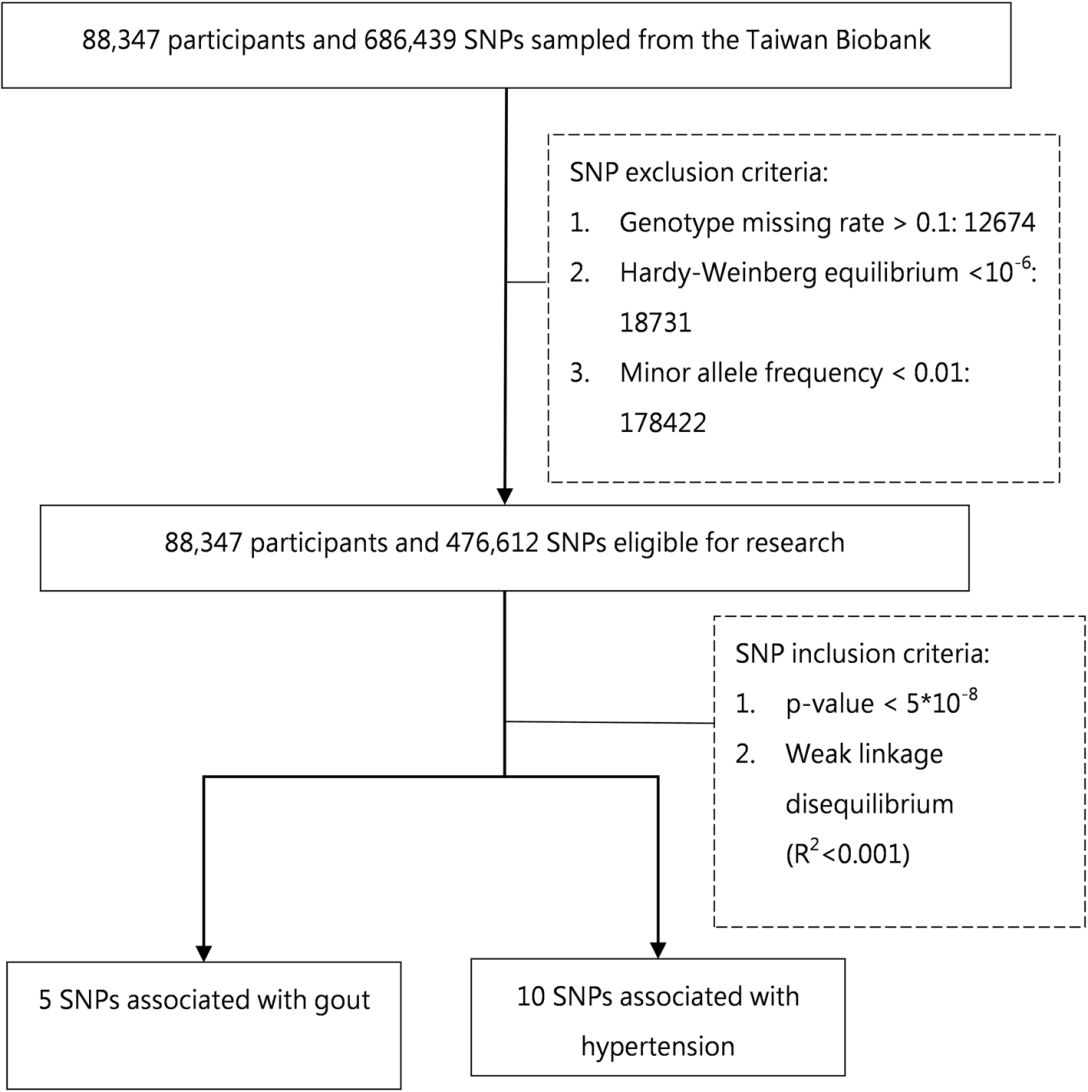
Flow chart of SNP and patient quality control. Parameters are listed in the chart. SNPs, single-nucleotide polymorphisms

A standard quality control (QC) procedure and genome-wide association studies (GWASs) were conducted by PLINK 1.9 (http://www.cog-genomics.org/plink/1.9/) [23]. For the QC procedure, we excluded SNPs with a low call rate (genotype missing rate > 0.1), a *p*-value for the Hardy–Weinberg equilibrium test of < 1.0 × 10^−6^ for controls, and a minor allele frequency of < 0.01. The Hardy–Weinberg equilibrium test offers additional support to confirm whether SNPs were randomly distributed, in a manner analogous to randomised controlled trials. For the GWAS procedure, we selected significant SNPs associated with gout or hypertension at a threshold *p*-value of < 5.0 × 10^−8^ in logistic regression analyses in three scenarios: unadjusted, adjusted to age and sex, and adjusted to age, sex, and BMI. For evaluation of linkage disequilibrium coefficients and haplotype association statistics, we used Haploview version 4.2 software (Mark Daly, Massachusetts Institute of Technology/Harvard Broad Institute, Cambridge, MA, USA) and LDLinkR [24], and then removed SNPs with high linkage disequilibrium (LD) with the correlation coefficient ${r}^{2}$ greater than or equal to 0.001. After these procedures, we obtained five SNPs associated with gout and ten SNPs related to hypertension, which were subsequently used as genetic IVs in this study. The SNPs selected were identical in the three scenarios during the GWAS procedure, which includes adjusting to different sets of covariates (Supplementary Fig. 1). To avoid biased results caused by pleiotropy, we used the PhenoScanner version 2 database (http://www.phenoscanner.medschl.cam.ac.uk/) [25, 26] to examine GWAS results from the Taiwan Biobank to identify any pleiotropic effects of the five SNPs of gout and ten SNPs of hypertension. The PhenoScanner version 2 database is publicly available and contains several results from large-scale genetic association studies in humans. This database includes over 150 million unique genetic variants, as well as more than 65 billion genotype–phenotype associations. After QC, we selected five SNPs associated with gout and ten associated with hypertension. Details of the selected SNPs are presented in Supplementary Table 1. The QC process for SNPs and patients is shown in Fig. 1. It should be noted that several of the SNPs associated with gout are also associated with serum urate. However, it is reasonable as the development of gout relies heavily on serum urate.

### Bidirectional MR analysis with coarsened exposures

MR is an analytical method that manages the causal structure that involves unmeasured confounders by using genetic variants as IVs to investigate causal effects from the exposure to the outcome. There are three core assumptions in conventional MR for genetic variants to be valid IVs: relevance, the genetic variants are predictive of the exposure; exogeneity, the genetic variants are independent of any unmeasured confounders of the exposure-outcome association under the condition on all measured confounders; and exclusion restriction, the genetic variants are conditionally independent of the outcome, considering the exposure and all confounders.

**Fig. 2:**
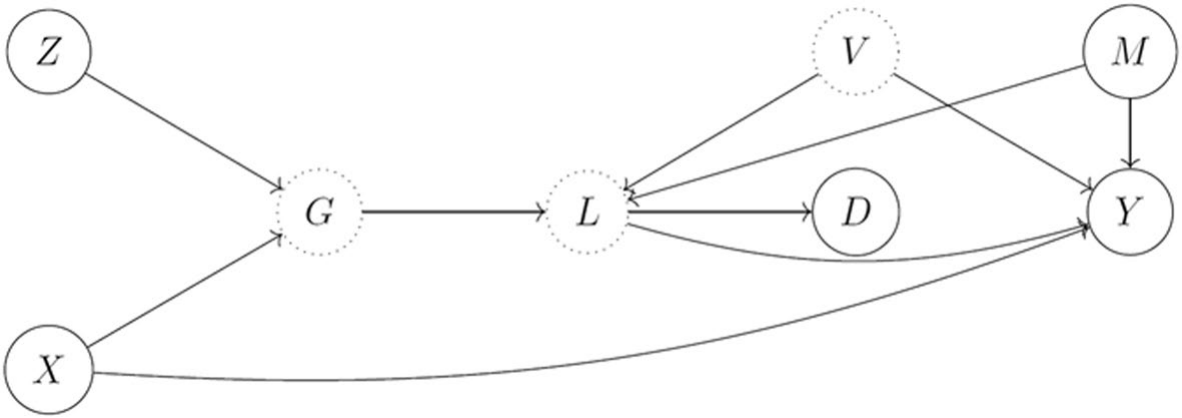
Directed acyclic graph for the MR with coarsened exposure framework under modified assumptions. Dotted circles represent unobserved variables and complete circles represent observed variables.$Z,$ multiple genetic IVs;$X,$ other genetic variables;$V,$ environmental share;$G,$ genetic share;$M,$ measured confounders;$L,$ latent exposure;$D,$ measured discrete exposure;$Y,$ continuous or binary outcome

Conventional MR analysis requires that the effects of exposure on the outcome and the genetic variants’ associations with the risk factors/outcome be linear without effect modification. The MR methods supporting binary or dichotomous exposure have not been clarified, despite IV analysis for continuous exposure/outcome and potential strategies to overcome the linearity assumption [27]. The application of conventional MR analysis to dichotomous exposures can result in unidentifiable relative risk (RR) values, in which the boundaries can be identified but not the exact causal effect. However, many clinically significant variables, such as the diagnosis of diseases, are binary. Therefore, we regarded the binary exposure as a coarsened approximation of a latent continuous trait, termed “coarsened exposure.” Although the genetic variant of coarsened exposure and the outcome may not be entirely mediated by the exposure, MR with coarsened exposures posed on the study by Tudball et al. [20] can handle this scenario and violation of the exclusion restriction assumption. To involve measured confounders and the binary outcome in this study, we consider “modified MR with coarsened exposures (See Fig. 2)” based on [20], which contain variables as follows: the coarsened exposure (binary) D, the liability L of D, the binary outcome Y, the genetic share of G, the environmental share V which is unmeasured, measured confounders M, multiple genetic IVs Z, other genetic variants X which can violate IV assumptions due to horizontal pleiotropy. We thus modified the latent variable approach mentioned in the work of MR with coarsened exposure [20] under the Falconer framework with modified assumptions to assess the causal effect between the liability of the exposure and the outcome. More detail of the modified MR with coarsened exposure are shown in Supplementary Methods 1, 2.

Furthermore, of the five SNPs selected as IVs for gout, one (rs671) may have pleiotropic effects on HTN, but in contrast, there were no such SNPs among the ten SNPs selected for hypertension for this study. SNP information are found in the “SNP selection” section and Supplementary Table 1.

### MR analysis with coarsened exposures using IVW estimators and PRSs

To estimate the causal effect of the latent variable of exposure on the outcome, we considered two standard methods, the IVW method and the PRS method (details are provided in Supplementary Methods 2.3–2.4) [28, 29]. For the IVW method, fixed-effect and random-effect will be determined based on Cochran’s Q test [30]. We used the R package *MendelianRandomization* for analysis [31]. Although the use of multiple IVs can improve estimate precision and provide a more narrow confidence interval [32], this method would be more susceptible to a biased result if some IVs are weak [33]. The Cragg–Donald F test is often used to assess IV strength; if the Cragg–Donald F test value is strictly less than 10, then the IV analysis may be susceptible to weak instrument bias [28]. However, the Cragg–Donald F test cannot be used on binary exposures or unobserved latent variables. Therefore, we implemented an alternative approach to ensure the strength of our selected IVs. We first used the likelihood ratio test (LRT) after maximum likelihood ratio estimation to determine whether the coefficients of the valid IVs are zero for the regression of the latent exposure on valid IVs. Importantly, a significant *p*-value obtained in LRT does not guarantee the strength of the genetic share and valid IVs; it increases the probability that the genetic share is composed of valid IVs. Moreover, to avoid biased results, we also considered another approach, using the PRS method to combine multiple genetic variants into a single score is a common approach for increasing IV strength [34]. The PRS can be used as a new IV to perform MR analysis. We evaluated the PRSs of gout and hypertension using five gout-associated SNPs and ten hypertension-associated SNPs, respectively. The weights were estimated from logistic regression using tenfold cross-validation because an independent dataset was unavailable [35].

### The parameter ${\theta }^{2}$ on modified MR analysis with coarsened exposures

In this MR with coarsened exposures structure, the parameter ${\theta }^{2}$ is defined as the fraction of latent phenotype variability that can be attributed to genetic variation or so-called heritability, and can be used as a sensitivity parameter to evaluate the causal effect over a range of ${\theta }^{2}$ values; it can be estimated using GWAS, as in the study by Lee et al. [36]. To verify the robustness of MR analysis results, we calculated the causal effect estimate over multiple ${\theta }^{2}$ values; the ceiling was determined by heritability (considering all possible genetic contributions) extracted from published studies. In particular, Nakatochi et al. demonstrated that the SNP-based heritability for urate was 0.14 [37], while Evangelou et al. showed that the SNP-based heritability for blood pressure was 0.21 [38]. We also verified ${\theta }^{2}$ values lower than our estimates from GWAS. Therefore, we assessed the RR effect of gout on hypertension using additional ${\theta }^{2}$ values of 0.04, 0.07, and 0.14, and additional ${\theta }^{2}$ values of 0.01, 0.07, 0.14, and 0.21 for the analysis of hypertension to gout.

This study conducted MR analyses with coarsened exposures under two different scenarios. First, we did not consider any measured confounders because MR analysis with coarsened exposures is analogous to a randomised controlled trial; therefore, it minimises bias from confounders. However, to ensure that confounders did not affect the results, we performed a second analysis using factors associated with gout and hypertension as measured confounders, including age, sex, BMI, and serum creatinine. We then performed the chi-squared test and Spearman rank correlation to evaluate whether the selected SNPs and measured confounders were independent, which corresponds to our modified assumption 5 in Supplementary Methods 2. These two tests were also used to assess multicollinearity for linear, probit, and log-linear regression analyses. The *p*-value was adjusted by controlling the false discovery rate. MR analysis with coarsened exposures yielded a more accurate RR with binary exposures than did conventional MR analysis methods; therefore, this modified approach would be more beneficial when discussing clinical problems.

## Results

### Baseline cohort descriptions

Table 1 shows the baseline characteristics of the 88,347 participants, among which 3253 (3.68%) had gout and 11,948 (13.52%) had hypertension. The mean age of the population was 51.1 years. Participants with gout or hypertension were more likely to be older and male, with a higher BMI and urate level, as well as worse kidney function.

**Table 1:** Baseline characteristics of the initial cohort

		Overall (*n* = 88,347)	Gout		Hypertension	
			Gout (*n* = 3253)	No gout (*n* = 85,094)	Hypertension (*n* = 11,948)	No hypertension (*n* = 76,399)
Age (years), mean (SD)		51.10 (11.12)	54.37 (10.70)	50.98 (11.12)	58.94 (8.22)	49.88 (11.02)
≥ 60		24,425 (27.6%)	1271 (39.1%)	23,154 (27.2%)	6471 (54.2%)	17,954 (23.5%)
50–59		26,253 (29.7%)	938 (28.8%)	25,315 (29.7%)	3902 (32.7%)	22,351 (29.3%)
40–49		20,016 (22.7%)	648 (19.9%)	19,368 (22.8%)	1248 (10.4%)	18,768 (24.6%)
30–39		17,653 (20.0%)	396 (12.2%)	17,257 (20.3%)	327 (2.7%)	17,326 (23.5%)
Female		60,157 (68.1%)	379 (11.7%)	59,778 (70.2%)	6585 (55.1%)	53,572 (70.1%)
BMI (kg/m^2^), mean (SD)		24.25 (3.83)	26.86 (4.02)	24.15 (3.79)	26.42 (4.02%)	23.91 (3.69%)
> 35		1098 (1.2%)	127 (3.9%)	971 (1.1%)	385 (3.2%)	713 (0.9%)
30–35		5508 (6.2%)	482 (14.8%)	5026 (5.9%)	1579 (13.2%)	3929 (5.1%)
27–30		11,635 (13.2%)	765 (23.5%)	10,870 (12.8%)	2630 (22.0%)	9005 (11.8%)
24–27		23,705 (26.8%)	1124 (34.6%)	22,581 (26.5%)	3931 (32.9%)	19,774 (25.9%)
18.5–24		43,425 (49.2%)	745 (22.9%)	42,680 (50.2%)	3350 (28.1%)	40,075 (52.5%)
< 18.5		2949 (3.3%)	10 (0.3%)	2939 (3.5%)	64 (0.5%)	2885 (3.8%)
Missing		27	0	27	9	18
Smoking						
Current smoker		7480 (8.5%)	584 (18.0%)	6896 (8.1%)	1087 (9.1%)	6393 (8.4%)
Stopped smoking		8747 (9.9%)	874 (26.9%)	7873 (9.3%)	2005 (16.8%)	6742 (8.8%)
Never or seldom		121 (0.1%)	14 (0.4%)	107 (0.1%)	23 (0.2%)	98 (0.1%)
Missing		71,997 (81.5%)	1781 (54.7%)	70,216 (82.5%)	8833 (73.9%)	63,164 (82.7%)
Drinking						
Current drinking		5079 (5.7%)	463 (14.2%)	4616 (5.4%)	981 (8.2%)	4098 (5.4%)
Stopped drinking		2399 (2.7%)	294 (9.0%)	2105 (2.5%)	652 (5.5%)	1747 (2.3%)
Seldom or never		80,840 (91.5%)	2496 (76.7%)	78,344 (92.1%)	10,311 (86.3%)	70,529 (92.3%)
Missing		22	0	22	3	19
Creatinine (mg/dL), mean (SD)		0.71 (0.31)	0.99 (0.55)	0.70 (0.29)	0.82 (0.53)	0.70 (0.25)
Urate (mg/dL), mean (SD)		5.35 (1.40)	7.27 (1.85)	5.27 (1.33)	5.95 (1.50)	5.25 (1.36)
Triglyceride (mg/dL), mean (SD)		117.27 (97.29)	168.91 (145.63)	115.30 (94.40)	144.06 (113.23)	113.08 (93.87)
Total cholesterol (mg/dL), mean (SD)		196.58 (36.23)	193.35 (36.88)	196.70 (36.20)	190.94 (36.57)	197.46 (36.10)
HDL (mg/dL), mean (SD)		55.10 (13.60)	46.66 (11.06)	55.42 (13.59)	50.27 (12.24)	55.86 (13.65)
LDL (mg/dL), mean (SD)		120.85 (31.91)	120.16 (32.84)	120.87 (31.88)	116.59 (32.04)	121.51 (31.84)

Data are shown as *n* (%) unless otherwise indicated. *Abbreviations*: *SD* Standard deviation, *BMI* Body mass index, *HDL* High-density lipoprotein, *LDL* Low-density lipoprotein

### MR analysis with coarsened exposures for gout on hypertension

**Fig. 3:**
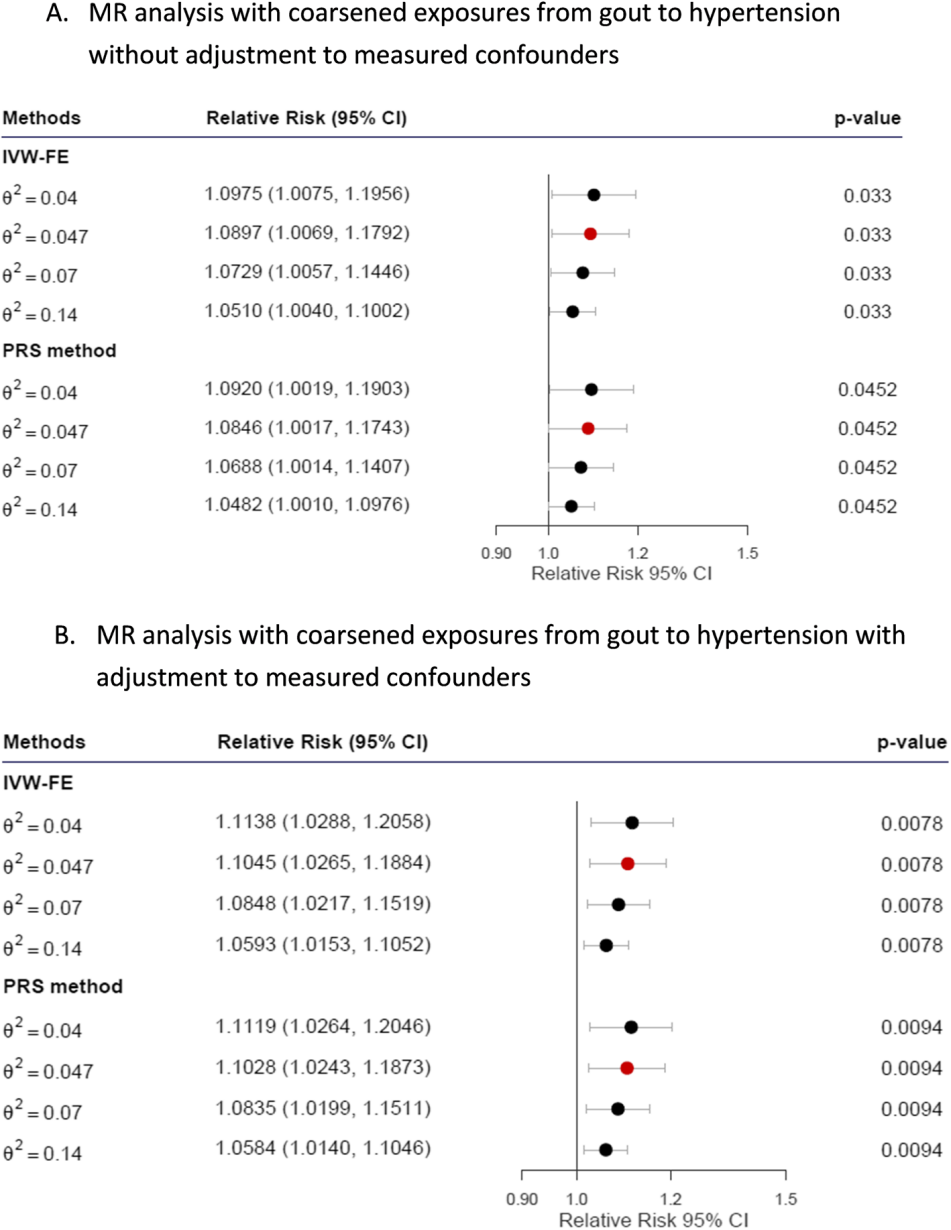
Plot of the relative risk of gout to hypertension. Relative risks calculated using the corresponding ${\theta }^{2}$ values estimated by our GWAS reports are marked in red. Abbreviations: IVW-FE, IVW fixed-effect method; PRS, polygenic risk score; CI, confidence interval

The results of MR analysis with coarsened exposures for gout on hypertension are presented in Fig. 3. We selected four gout-associated SNPs as valid IVs (as a role of Z in Fig. 2, also see Supplementary Table 1.1), and one SNP (rs671 are as a role of X in Fig. 2, also see Supplementary Table 1,2) may have pleiotropic effects on HTN, for MR analysis with coarsened exposures. When these five SNPs were combined as the genetic share, the estimated ${\theta }^{2}$ values for gout-associated SNPs were 0.047. The LRTs for gout-associated SNPs were 832.16 (*p* < 2.2 × 10^−16^) before and 892.41 (*p* < 2.2 × 10^−16^) after adjusting to measured confounders, supporting the credibility of the genetic share composed of the selected SNPs. The Cochran’s Q test for gout-associated SNPs before adjusting to measured confounders was 3.42 (*p* = 0.3314), suggesting that the effects of gout-associated SNPs were homogenous, and the fixed-effect IVW method is more suitable than the random-effects method. At the estimated ${\theta }^{2}$, the RRs were 1.09 (95% confidence interval [CI], [1.01–1.18], *p* = 0.033) for the fixed-effect IVW method and 1.08 (95% CI, [1.00–1.17], *p* = 0.0452) for the PRS method. After adjustments for measured confounders, Cochran’s Q test continued to support homogenous effects of the selected SNPs (5.22, *p* = 0.1566); the results remained significant for the fixed-effect IVW method (RR, 1.10; 95% CI, [1.03–1.19]; *p* = 0.0078) and the PRS method (RR, 1.10; 95% CI, [1.02–1.19], *p* = 0.0094). Collectively, the results support the causality of the liability of gout on hypertension.

### MR analysis with coarsened exposures for hypertension on gout

**Fig. 4:**
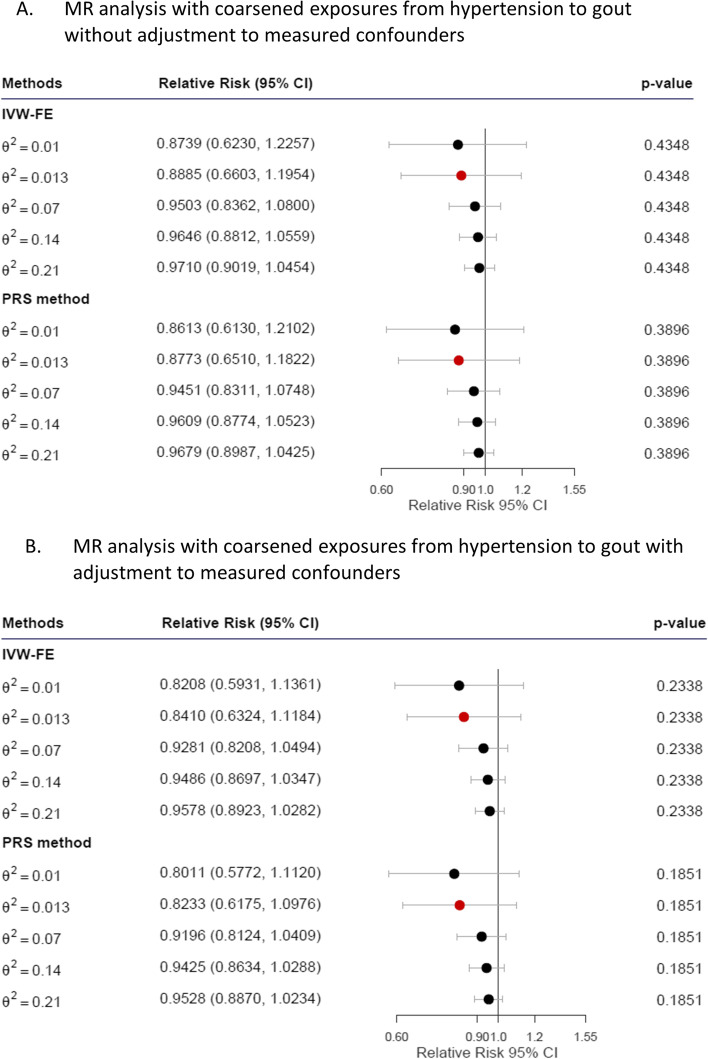
Plot of the relative risk of hypertension to gout. Relative risks calculated using the corresponding ${\theta }^{2}$ values estimated by our GWAS reports are marked in red. Abbreviations: IVW-FE, IVW fixed-effect method; PRS, polygenic risk score; CI, confidence interval

The results of the MR analysis with coarsened exposures for hypertension on gout are shown in Fig. 4. We selected ten hypertension-associated SNPs (see Supplementary Table 1.3) as IVs to examine the causal effect of the liability of hypertension on gout. The LRTs for hypertension-associated SNPs were 461.50 (*p* < 2.2 × 10^−16^) and 514.63 (*p* < 2.2 × 10^−16^) before and after adjustment to measured confounders, supporting the credibility of the genetic share being composed of the selected valid IVs. The estimated values of ${\theta }^{2}$ for hypertension-associated SNPs were 0.013. Before adjustment to measured confounders, Cochran’s Q test value for the ten hypertension-associated SNPs was 14.08 (*p* = 0.1194), suggesting homogenous effects of SNPs; hence, the fixed-effect method was applied. After adjustments for measured confounders, Cochran’s Q test value remained nonsignificant (15.91, *p* = 0.0688). At the specified ${\theta }^{2}$, RRs were not significant for both fixed-effects IVW and PRS models, regardless of adjustment. The results remained nonsignificant across a range of ${\theta }^{2}$ values; therefore, they did not support a causal effect of hypertension on gout.

### Modified MR with coarsened exposures under removed SNPs associated exposure and measured confounders

**Fig. 5:**
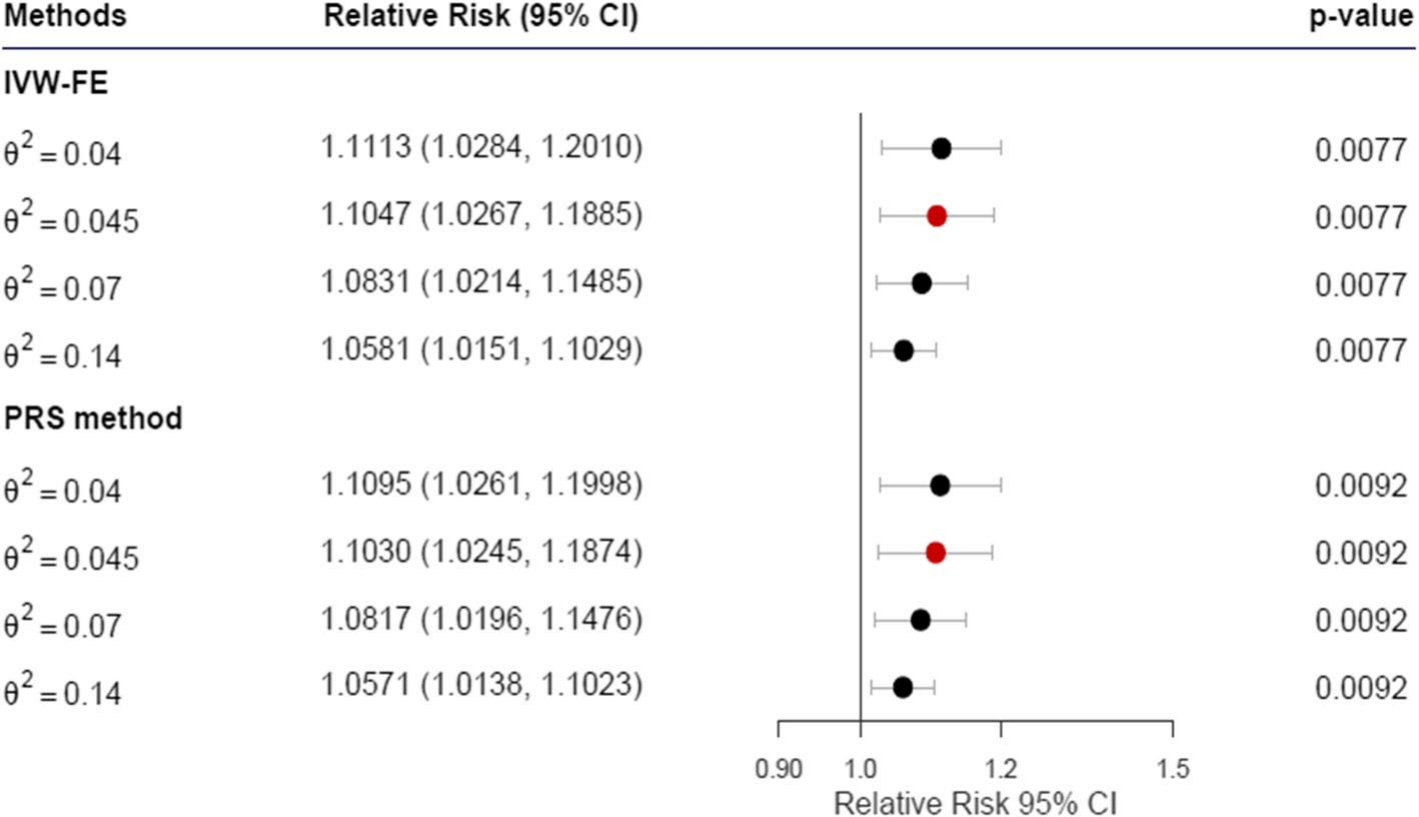
MR analysis with coarsened exposures from gout to hypertension with adjustment to measured confounders after removing SNPs associated with measured confounders. IVW-FE, IVW fixed-effects method; PRS, polygenic risk scores; CI, confidence interval

To evaluate modified assumption 5 of modified MR with coarsened exposure (the genetic variants should be independent with measured confounder) and multicollinearity, we performed the chi-square test of independence and Spearman correlation tests between selected SNPs and measured confounders. Most of the selected SNPs were independent of all measured confounders, and only a few SNPs were associated with creatinine (See Supplementary Table 2). Therefore, the selected SNPs did not severely violate this assumption, and only small amounts of multicollinearity were observed in our model. To further validate the results, we removed SNPs with a *p* < 0.05, which resulted in only one SNP rs671 associated with gout have been excluded in conducting an MR analysis with coarsened exposures to evaluate the causal effects of the liability of gout on hypertension, including measured confounders (Supplementary Table 3). Although we think SNP rs671 (as role X in Fig. 2) does not affect the causal estimate much and still conducted an MR analysis with coarsened exposures. The result of the LRTs and Cochran’s Q test for gout-associated SNPs, and compared with previous results, are present in Table 2. The new ${\theta }^{2}$ value after removal of rs671 is 0.45, and the LRT value is 862.65 (*p*-value < 2.22 × 10^−16^), and the Cochran’s Q test was 5.22 (*p*-value 0.1563), supporting the application of the fixed-effect method. The results remained significant after adjustment for measured confounders for the fixed-effect IVW method (RR, 1.10; 95% CI, [1.03–1.19]; *p* = 0.0077) and the PRS method (RR, 1.10; 95% CI, [1.02–1.19], *p* = 0.0092). The results are shown in Fig. 5 below. Additional results for no involved measured confounders and removing SNPs rs671 can be founded in Supplementary Fig. 2.

**Table 2:** The LRT, Cochran’s Q test, and ${\theta }^{2}$ in MR analysis with coarsened exposures for gout on hypertension under different scenarios

Remove SNPs?^a^	Measured confounders?	LRT (*p*-value)	Cochran's Q test (*p*-value)	${\uptheta }^{2}$
No	Yes	892.4092 (< 2.22 × 10^−16^)	5.2169 (0.1566)	0.047
No	No	832.1562 (< 2.22 × 10^−16^)	3.4188 (0.3314)	0.047
Yes	Yes	862.6535 (< 2.22 × 10^−16^)	5.2209 (0.1563)	0.045
Yes	No	804.67 (< 2.22 × 10^−16^)	3.4271 (0.3303)	0.045

^a^Remove SNP associated with measured confounders

On the other hand, the results for HTN on gout are exactly the same as before because no SNPs associated with HTN have been excluded (See Supplementary Tables 2 3, and Fig. 4). These analyses yielded similar results as before: a significant effect of the liability of gout on hypertension but not of hypertension on gout.

## Discussion

We used a modified MR with coarsened exposures to investigate the bidirectional causal effects between the liability of gout and hypertension with data from the Taiwan Biobank. The results indicate that the liability of gout is causal to hypertension, but hypertension is not causal to gout. Furthermore, the robustness of the results obtained using multiple models with covariate adjustments strengthens the conclusion.

Many studies have investigated the relationship between serum urate and hypertension [39, 40]. A meta-analysis of the results of 18 prospective studies with 55,607 total participants reported that an elevated serum urate level was associated with an adjusted RR of 1.41 for incident hypertension [41]. An association between gout and hypertension has long been observed [42, 43]. In the Normative Aging Study, gout was threefold more common among hypertensive participants than among non-hypertensive participants [44]. Riedel et al. found that the prevalence of hypertension was 60.9% in 9482 gout patients [45]. In a large US cohort with 5942 gout patients, the prevalences of hypertension were 36.2% in incident gout patients and 45.7% in prevalent gout patients [46]. Individuals with hypertension are generally twice as likely to develop incident gout [47]. Despite solid epidemiological evidence to support an association between gout and hypertension, the causality and direction of the association are unclear.

Recent MR studies have used genetic risk scores calculated from specific SNPs or have used SNPs selected from the literature, as IVs to investigate the causal effect of urate on blood pressure [16–19]; however, the results have been conflicting. Some studies using the IVW method suggested that urate elevated blood pressure [16, 18], but they discovered pleiotropic effects that affected the interpretation of the results. Other studies suggested that urate may not be associated with hypertension [17, 19]. While two studies noted pleiotropic SNPs that may affect their interpretation of the causal effect of serum urate on blood pressure,[16, 18], the SNPs used in those studies differed from those used in this study: SLC2A9 and ABCG2 genes are known urate genes that are the leading loci driving the association with gout, and we find SNP rs2231142 at ABCG2 is considered both in their and our study. However, the SNPs in SLC2A9 genes are different. In addition, the SNPs having a pleiotropy effect on blood pressure mentioned in the aforementioned studies [16, 18] did not overlap with the SNP associated with gout in our study. We also checked through the PhenoScanner to identify pleiotropic SNPs. Finally, the exposure used in those studies is serum urate, whereas this study used gout. Although an elevated urate level is a risk factor for gout, hyperuricaemia does not necessarily imply gout [48]. Richette et al. hypothesised that gout might increase cardiovascular risk through chronic inflammation caused by monosodium urate crystal disposition [49]. Gout and urate should be viewed as different conditions, and the associated SNPs and systemic effects differ. It should be noted that while gout is not equivalent to hyperuricaemia, elevated serum urate plays an important role in the pathogenesis and liability of gout, and assigning serum urate as a confounder would then remove a significant portion within the liability of gout. To our knowledge, this is the first MR study to assess the causal relationship between the liability gout and hypertension.

This MR study used coarsened exposures to evaluate the causal effect of the liability of gout on hypertension. Although both hyperuricaemia and gout have been associated with hypertension, it remains unknown whether gout as an index disease causes hypertension. The case–control study by Sandoval-Plata et al. showed that gout is associated with hypertension after adjustment for serum urate [3], suggesting an alternative pathway from gout to hypertension other than via hyperuricemia. The present study supported a causal effect of gout on hypertension, but multiple MR analyses have not provided conclusive evidence to support a direct causal effect of the urate level on hypertension [16–19]. Therefore, the causal effect of gout on hypertension may be mediated by the urate level, in combination with other urate-independent pathways.

Most observational studies show that hypertension is associated with gout. For example, a UK study based on primary care data found that gout patients were twofold more likely than controls to have hypertension at diagnosis [1]. However, the present study found no evidence that hypertension causes gout, suggesting that the association between hypertension and gout may not be caused directly by hypertension; it may be mediated by other pathways. Several medications increase the risk of gout by increasing the serum urate level by reducing renal urate excretion. Diuretics are the most commonly encountered drug in this respect [50]. Therefore, hypertension is associated with gout, but it is not causal of gout.

The strengths of this study include the use of the Taiwan Biobank and a novel MR analysis method to investigate causal effects through dichotomous exposures. By using the Taiwan Biobank as a data source, we ensured sufficient sample size and proper genotyping/phenotyping to examine the causal relationship between gout and hypertension. Additionally, we implemented MR analysis with coarsened exposures. To our knowledge, this study is among the earliest to use this approach for a medical analysis. This novel study design also enabled us to apply the MR model for causal inferences regarding binary exposures such as gout, then obtain an accurate causal effect size.

There were several limitations in this study. Although MR analysis with coarsened exposures can use SNPs with pleiotropic effects to increase the efficiency of estimates, incorrect classification of pleiotropic SNPs as valid IVs may have introduced biased results. To counteract this problem, we used the PhenoScanner database to search for possible pleiotropic effects of SNPs. Because the Taiwan Biobank uses questionnaires for the classification of gout and hypertension, there may have been recall bias that affected interpretation of the results. Furthermore, we only assessed the Taiwanese population, which may have different epidemiological or genetic features concerning gout and hypertension, compared with other Asian populations [22]. Further studies in other populations worldwide are warranted to assess the generalizability of our findings.

## Conclusions

Modified MR with coarsened exposures model revealed a robust causal role of the liability of gout in hypertension onset but not a causal role of hypertension in gout onset. Thus, it may be beneficial for clinicians to view gout as a chronic disease with important systemic effects rather than simply acute self-limiting arthritis.

### Software and package

The code for modified MR with coarsened exposure in this study is mainly based on [21] (https://github.com/matt-tudball/mrlat_replication) and [31] (R package *MendelianRandomization)*. R version(4.0.3), LDLinkR (https://github.com/CBIIT/LDlinkR) [24], PLINK 1.9 (http://www.cog-genomics.org/plink/1.9/) [23], and PhenoScanner version 2 database (http://www.phenoscanner.medschl.cam.ac.uk/) [25, 26]. The code for modified MR with coarsened exposure can be founded in the file: Supplementary code_ modified MR with coarsened exposures.txt.

## Supplementary Information

**Additional file 1**: Supplementary methods: Assumptions and mathematical proof for the modified MR with coarsened exposure.**Supplementary Table 1. ** Detailed information on SNPs associated with gout and hypertension contains SNPs associated with gout, heterogeneous SNPs associated with gout, and SNPs associated with HTN. **Supplementary Table 2. ** P-values for the association between SNPs and measured confounders. **Supplementary Table 3**. Comparing no remove and remove SNPs associated with exposure correlated with measured confounders. **Supplementary Figure 1**: Flow chart for GWAS procedure of SNP selection under three different settings. **Supplementary Figure 2**. The results of MR analysis without coarsened exposures after removing SNPs associated without measured confounders. Supplementary Code: Modified MR with coarsened exposures.

## Data Availability

The dataset supporting the conclusion of this article is available in the Taiwan Biobank (https://www.twbiobank.org.tw/new_web/) upon reasonable request.
