# Supplementary material for: Assessing the causal relationships between gout and hypertension: a bidirectional Mendelian randomisation study with coarsened exposures
Source: Arthritis Res Ther. 2022 Oct 29;24:243. doi: 10.1186/s13075-022-02933-4 (PMC9617405; doi:10.1186/s13075-022-02933-4)
Supplement: Supplementary file 1 — Additional file 1: Supplementary methods: Assumptions and mathematical proof for the modified MR with coarsened exposure.Supplementary Table 1. Detailed information on SNPs associated with gout and hypertension contains SNPs associated with gout, heterogeneous SNPs associated with gout, and SNPs associated with HTN. Supplementary Table 2. P-values for the association between SNPs and measured confounders. Supplementary Table 3. Comparing no remove and remove SNPs associated with exposure correlated with measured confounders. Supplementary Figure 1: Flow chart for GWAS procedure of SNP selection under three different settings. Supplementary Figure 2. The results of MR analysis without coarsened exposures after removing SNPs associated without measured confounders. Supplementary Code: Modified MR with coarsened exposures. [file 13075_2022_2933_MOESM1_ESM.docx]

Content of Supplementary

[**Supplementary Methods: Mendelian Randomization with coarsened exposures** 1](#_Toc112925288)

[**1. Original assumptions for MR with coarsened exposures** 1](#_Toc112925289)

[**2. Modified MR analysis with coarsened exposures** 2](#_Toc112925290)

[**2.1 Modified assumptions for MR with coarsened exposure** 2](#_Toc112925291)

[**2.2 Latent variable approach under modified assumptions** 3](#_Toc112925292)

[**2.3 Inverse-variance weighted estimators** 6](#_Toc112925293)

[**2.4 Polygenic risk score calculation with k-folds cross-validation** 7](#_Toc112925294)

[**Supplementary Tables** 8](#_Toc112925295)

[**Table 1. Detailed information on SNPs** 8](#_Toc112925296)

[**Table 1.1 SNPs associated with gout** 8](#_Toc112925297)

[**Table 1.2 Heterogeneous SNPs associated with gout** 10](#_Toc112925298)

[**Table 1.3 SNPs associated with HTN** 12](#_Toc112925299)

[**Table 2. P-values for the association between SNPs and measured confounders.** 20](#_Toc112925300)

[**Table 3. Comparing no remove and remove SNPs which associated with exposure correlated with measured confounders** 21](#_Toc112925301)

[**Supplementary Figures** 22](#_Toc112925302)

[**Figure 1**. **Flow chart for GWAS procedure of SNP selection** 22](#_Toc112925303)

[**Figure 2**. **MR analysis with coarsened exposures from gout to hypertension without measured confounders after removing SNPs associated with measured confounders.** 23](#_Toc112925304)

[**References** 23](#_Toc112925305)

**Supplementary Methods: Mendelian Randomization with coarsened exposures**

**1. Original assumptions for MR with coarsened exposures**

Since conventional Mendelian randomization (MR) analysis may produce inflated or deflated results when applying categorical variables as exposures, a modified MR model (i.e., MR with coarsened exposures) is used to apply MR-based methods to dichotomous exposures.[1] The MR with coarsened exposures model uses the latent variable approach to estimate the causal effect from the latent continuous variable of the dichotomous exposures on the outcome; it is based on the Falconer framework, which assumes that $L$ represents disease liability and the dichotomous exposure $D$ can be categorized using the latent continuous exposure $L$ and a threshold value. There are several assumptions for the original framework[1]:

1. Single threshold: The relationship between the latent continuous exposure $L$ and the dichotomous exposure $D$ can be defined as $D=I \{L\geq0\}$, where $I$ is a characteristic function.
2. Additivity: The latent exposure $L=G+V$, where $G$ is the genetic share and $V$ is the environmental share of $L$.
3. Linearity: Consider $Z$ represents instrumental genetic variables that satisfy IV1 and IV2 assumptions and have no pleiotropic effects on the outcome; X are other genetic variants that satisfy IV1 and IV2 assumptions and have uncorrelated pleiotropic effects on the outcome. Genetic shares can be identified as follows: $G=\mu+\alpha Z+\gamma^{T}X$ for some scalar number $\alpha$ and scalar vector$\gamma$. Note that $Z$ violates the exclusion restriction assumption. For convenience, we also denote Z as valid IVs in this study.
4. Environmental share: The environmental share $V$ has a mean of zero, a standard error of $\sigma_{V}$And is in a family of continuous distributions; its cumulative distribution function is defined as $F(\nu/\sigma_{{}_{V}})=F_{V}(\nu)$ and density $f(\nu/\sigma_{{}_{V}})=f_{V}(\nu)$.
5. Risk factor independence:$Z$, $X$, $M$ and $V$ are mutually independent.
6. Gene-environment equivalence: The continuous outcome model assumes the form $Y=\beta L+\varepsilon_{{}_{X,V}}$, where $\beta$ is the causal effect of L on the continuous outcome $Y$, and $\varepsilon_{{}_{X,V}}$ is a random disturbance that may depend on $X$ and $V$.
7. IV assumptions: $Z=\{Z_{j}{\}}_{j=1}^{J}$ is independent of $\varepsilon_{{}_{X,V}}$, and the association of IVs, and the exposure is not zero.

**2. Modified MR analysis with coarsened exposures**

**2.1 Modified assumptions for MR with coarsened exposure**

In this study, we modified some assumptions regarding the original framework of MR with coarsened exposures, such that the redefined framework contains multiple IVs and measured confounders. In the additivity assumption, we considered the measured confounder $M=\{M_{i}{\}}_{i=1}^{m}$ with its associated coefficient $\xi=\{\xi_{i}{\}}_{i=1}^{m}$And modified the original relationship to the latent exposure $L$ is represented by: $L=G+\left( V+\sum_{i=1}^{m} \xi_{i}M_{i} \right)$. In the risk factor independence assumption:$Z$, $X$, $M,$ and $V$ are mutually independent. In the linearity assumption, we modified $Z$ to represent multiple genetic IVs; the linear function $G$ was redefined as$G=\mu+\alpha^{T}Z+\gamma^{T}X$, for some scalars vectors $\alpha$ and $\gamma$. In the gene-environment equivalence assumption, the continuous outcome model is $Y=\beta L+\varepsilon_{{}_{X,V}}$Whereas the binary outcome model is $log Y=\beta L+\varepsilon_{{}_{X,V}}$. Finally, for the IV assumptions, all IVs $Z=\{Z_{j}{\}}_{j=1}^{J}$ were independent of $\varepsilon_{{}_{X,V}}$; the associations between IVs and exposure must not be zero. The Modified assumptions for MR with coarsened exposure are as follows:

| 1. Single threshold: The binary measurement D and the latent exposure L are related by the single threshold model, as in the equation $D=I \{L\geq0\}$. |
| --- |
| 2. Additivity: The latent exposure $L$ is represented by:  $L=G+ (V+\sum_{i=1}^{m} \xi_{i}M_{i}$),  where $G$ is the genetic share and $V$ is the environmental share of $L$; $M$= $\left\{ M_{i} \right\}_{i=1}^{m}$ is the measured confounder, and it's coefficient$\xi$= $\left\{ \xi_{i} \right\}_{i=1}^{m}$. |
| 3. Linearity: Consider $Z$ to represent multiple genetic IVs that satisfy the IV1 and IV2 assumptions and have no pleiotropic effects on the outcome, while $X$ represents other genetic variants that satisfy IV1 and IV2 assumptions but have pleiotropic effects on the outcome. Then, we can define the genetic share as follows: $G=\mu+\alpha^{T}Z+\gamma^{T}X$, for some scalars vectors $\alpha$ and $\gamma$. Note that, although $Z$ violates the exclusion restriction assumption, we denoted $Z$ as a valid IV for convenience. |
| 4. Environmental share: The environmental share $V$ has a mean of zero, a standard error of $\sigma_{V}$, is in a family of continuous distributions, and its cumulative distribution function is defined as $F(\nu/\sigma_{{}_{V}})=F_{V}(\nu)$ and density function $f(\nu/\sigma_{{}_{V}})=f_{V}(\nu)$. |
| 5. Risk factor independence:$Z$, $X$, $M$, and $V$ are mutually independent. |
| 6. Gene-environment equivalence: The continuous outcome model assumes the form $Y=\beta L+\varepsilon_{{}_{X,V}}$; the binary outcome model is modified as $log Y=\beta L+\varepsilon_{{}_{X,V}}$, where $\beta$ is the causal effect of L on the continuous outcome $Y$, while $\varepsilon_{{}_{X,V}}$ is a random disturbance that may depend on $X$ and $V$. |
| 7. IV assumptions: $Z$ is independent of $\varepsilon_{{}_{X,V}}$; the associations of all IVs and the exposure are not zero. |

**2.2 Latent variable approach under modified assumptions**

To estimate the causal effect $\beta$ of the latent exposure $L$ of dichotomous exposures $D$ on the continuous or binary outcome $Y$, we used the modified latent variable approach. We began with the modified critical Lemma, such as Appendix A in the original latent variable approach.[1]

**Lemma**. *Given the observed data* $(Z,X,D,Y,M)$*,* $V$ *is a continuous random variable and* $\psi$ *is a continuous function on* $(Z,X,M)$*. Consider the linear operator* $L$*, given by* $L=\psi\left( Z,X,M \right)+V$*, and the single threshold model* $D=I \{L\geq0\}$*. The observable joint distribution of* $(Z,X,D,Y,M)$ *is consistent with any monotone transform of* $\psi(Z,X,M),$ *including* $P(D=1|Z,X,M).$

*Proof.* Since the cumulative distribution of $V,$ $F_{V}$ is a monotone function, $D$is invariant to $F_{V}$ in the sense that:

$$D=I\{\psi(Z,X,M)\geq V\}=I\{F_{V}(\psi(Z,X,M))\geq F_{V}(V)\}.$$

Considering that $V$ is a continuous random variable and $F_{V}$ is a monotone function, by basic theory, we find that $F_{V}$ has a uniform distribution on $(0,1).$ We denoted $F_{V}$ by $U\sim Unif(0,1)$. Moreover,

$$F(\psi(Z,X,M))=Pr(V\leq\psi(Z,X,M))=Pr(D=1|Z,X,M)$$

This implies:

$$D=I\{\psi(Z,X,M)\geq V\}=I\{Pr(D=1|Z,X,M)\geq U\}.$$

We used the same argument as the proof in Appendix A.[1] We found that the observable joint distribution of $(Z,X,D,Y,M)$ is consistent with any monotone transform of $\psi(Z,X,M).$ Thus, we complete the argument of this proof.

**Remark.** For technical reasons and the additivity assumption in the framework, we consider $D^{'}$ to be a random variable defined by $D^{'}=1-D$; we applied this critical Lemma to redefined observed data $\left( Z,X,D^{'},Y,M \right)$ in the proof of the Main theorem, not the original data$\left( Z,X,D,Y,M \right).$

**Main Theorem.** *Consider the Falconer framework under modified assumptions. Given the observed data* $(Z,X,D,Y,M)$ *and the latent exposure* $L$ *in this framework, consider that its standard error of* $\sigma_{L}^{2}$ *is given by* $\sigma_{L}^{2}=\sigma_{G}^{2}+\sigma_{V}^{2}+\sum_{i=1}^{m} \xi_{i}^{2}\sigma_{M_{i}}^{2}.$*And the genetic variance of the latent exposure is given by* $\theta^{2}=\frac{\sigma_{G}^{2}}{\sigma_{L}^{2}}.$*For the appropriate choice of the genetic variance of the latent exposure* $\theta^{2}.$T*he causal effect* $\beta$ *from the latent exposure* $L$ *of dichotomous exposure* $D$ *on continuous or binary outcome* $Y$ *can be identified as* $\beta_{L}=\sigma_{L}\beta$*from the observed data* $(Z,X,D,Y,M).$

*Proof.* We followed the proof of the latent variable approach in the article by Tudball *et al*. [1] and then divided it into four steps.

Step 1. Estimate the linear predictor of the generalized model $D$ on $Z,$ $X,$ and $M$.

Recall that the latent exposure $L$ is represented by:

$$L=G+\left( V+\sum_{i=1}^{m} \xi_{i}M_{i} \right)$$

, where $G$ is the genetic share of $L$ and $V$ is the environmental share of $L$; $M=\{M_{i}{\}}_{i=1}^{m}$ is given measured confounders and its coefficients $\xi=\{\xi_{i}{\}}_{i=1}^{m}$. By the modified risk factor independence assumption, we know that $Z,M,X,$ and $V$ are mutually independent. Consider the new random variable $D^{'}=1-D$. This immediately gives:

$$P\left( X=x,Z=z,M=m \right) =P\left( X=x,Z=z,M=m \right) =P\left( X=x,Z=z,M=m \right) =P\left( V\geq-\left( \nu+\alpha^{T}z+\gamma^{T}x+\sum\xi_{i}m_{i} \right) \right) =1-F\left( {-\sigma}_{V}^{-1}\left( \nu+\alpha^{T}z+\gamma^{T}x+\sum\xi_{i}m_{i} \right) \right) =1-F\left( -\left( \tilde{\nu}+\tilde{\alpha}^{T}z+\tilde{\gamma}^{T}x+\sum\tilde{\xi}_{i}m_{i} \right) \right),$$

where the vector $\sigma_{V}^{-1}\cdot u$ is denoted by $\tilde{u}$ for any vector $u$. Therefore:

$$P\left( X=x,Z=z,M=m \right)= P\left( X=x,Z=z,M=m \right)$$

$=1-P\left( X=x,Z=z,M=m \right)$

$=F\left( -\left( \tilde{\nu}+\tilde{\alpha}^{T}z+\tilde{\gamma}^{T}x+\sum\tilde{\xi}_{i}m_{i} \right) \right)$

We took the case $\psi\left( Z,X,M \right)={-\sigma}_{V}^{-1}\left( G+\sum_{i=1}^{m} \xi_{i}M_{i} \right)$in **Lemma** above, then applied the Lemma to redefined observable data $(Z,X,D',Y,M)$. The joint distribution of $(Z,X,D',Y,M)$ is consistent with any monotone transform of $\psi\left( Z,X,M \right)$, implying that the original data $\left( Z,X,D,Y,M \right)$ also obey this relationship. We consider $F$ to have a normal distribution; thus, the parameters $\tilde{\nu},\tilde{\alpha},\tilde{\gamma},$ and $\tilde{\xi}_{i}$ can be estimated via maximum likelihood ratio estimation. Next, we used the parameters $\tilde{\nu},\tilde{\alpha},\tilde{\gamma}.$And observed data $(Z,X,M)$ to construct the predicted genetic share of the latent exposure:

$\tilde{G}=\tilde{\nu}+\tilde{\alpha}^{T}Z+\tilde{\gamma}^{T}X=\frac{G}{\sigma_{V}}$.

Notice that the predicted genetic share $\tilde{G}$ involved the standard error of the environmental share, which is unknown. Here, we used the likelihood ratio test (LRT) to determine whether the coefficients of the valid IVs $Z$ are zero for regression of the latent exposure on valid IVs. Notably, a significant p-value obtained in LRT does not guarantee the strength of the genetic share and valid IVs; however, it increases the likelihood that the genetic share is composed of valid IVs.

Step 2. Normalize the linear predictor to be independent of the standard error of environmental shares.

Again, we remove the standard error of unobserved environmental share $\sigma_{V}$, using the same argument as Step $2$ in the article by Tudball *et al*.[1] More precisely, we standardized $\tilde{G}$ by the standard error $\sigma_{\tilde{G}}$; this yields the random variable $G/\sigma_{G}$, which is not involved in the unobserved $\sigma_{V}$, and satisfies:

$$\frac{G}{\sigma_{G}}=\frac{\tilde{G}/\sigma_{V}}{\sigma_{\tilde{G}}/\sigma_{V}}=\frac{\tilde{G}}{\sigma_{\tilde{G}}},$$

Even though the genetic share is unobserved in general, such construction allows us to derive the exposure $G/\sigma_{G}$ as some substitute of the genetic share, which is the continuous random variable not involved unknown environmental share, totally estimated by maximum likelihood estimation from observed data. Thus, we further considered $G/\sigma_{G} as$the exposure and then conducted the IV analysis on it on the next step.

Step 3. Use the normalized linear predictor as the exposure in IV analysis.

Now, we conducted the IV analysis on the exposure $G/\sigma_{G}$. Under the condition of measured confounders, for the case that $Y$ is the continuous outcome, the estimate in IV analysis for each component $Z_{j}$can be described as follows:

$$\frac{cov(Z_{j},Y|M)}{cov(Z_{j},G/\sigma_{G}|M)}=\sigma_{G}\beta_{j}$$

For the case that $Y$ is a binary outcome, the ratio of the log-coefficient from a log-linear regression of the binary outcome $Y$ on the j*th* component of ${Z, Z}_{j}$, under the condition of $M$, and the coefficient from new exposure $G/\sigma_{G}$ on the j*th* component of ${Z, Z}_{j},$under the condition of $M$, is also equal to $\sigma_{G}\beta_{j}.$

Step 4. Scale the effect estimate up by the genetic variance of the latent exposure:

The final proof process is the same as Step 4 in the article by Tudball *et al*.[1] For the same reason, the resulting estimate in IV analysis in Step 3 is scaled by the standard of the genetic share; it is inconsistent with the causal effect of the liability $L$ of the dichotomous exposures $D$ on the outcome $Y$. Therefore, we used the genetic variance of the latent exposure $L$ given by:

$$\theta^{2}=\frac{\sigma_{G}^{2}}{\sigma_{L}^{2}},$$

which refers to the heritability of the liability as a parameter to obtain a causal effect estimate and the standard error of $\sigma_{L}^{2}$ is given by:

$$\sigma_{L}^{2}=\sigma_{G}^{2}+\sigma_{V}^{2}+\sum_{i=1}^{m} \xi_{i}^{2}\sigma_{m_{i}}^{2}.$$

This immediately implies the estimate:

$$\frac{\beta_{G}}{\theta}=\sigma_{L}\beta=\beta_{L}$$

, which is more typically the desired scale.

Moreover, $\theta^{2}$can be used as a sensitivity parameter to evaluate the causal effect over a range of$\theta^{2}$values, using GWAS reports, as described by Lee *et al*.[2] In this study, we estimated $\theta^{2}$ by deriving the coefficients of determination $R^{2}$ on the probit liability scale, $R_{probit}^{2}$:

$R_{probit}^{2}=\frac{var(\hat{b}_{probit}\times G)}{var\left( \hat{b}_{probit}\times G \right)+var\left( \frac{V}{\sigma_{v}} \right)}$,

where $\hat{b}_{probit}$is the estimated coefficient of the genetic share $G$ from a probit regression. The expectation of $R_{probit}^{2}$would be the heritability $\theta^{2}$, as above.

For the appropriate $\theta^{2}$of the genetic variants of latent exposure, the causal effect of the latent exposure of the dichotomous exposure on outcomes can be identified as a causal effect estimate that is scaled by the standard deviation of the latent exposure from the observed data. Thus, we complete the argument of this proof.

**2.3 Inverse-variance weighted estimators**

Instead of a single IV, we used multiple IVs to improve estimation efficiency. Suppose that the association estimates form a new exposure $G/\sigma_{G}$ on the j*th* component of ${Z, Z}_{j}$ under the condition $M$, denoted by $\hat{\beta}_{G_{j}}$. Furthermore, the log-coefficient from a log-linear binary outcome $Y$ is denoted by $\hat{\beta}_{Y_{j}}$ on the j*th* component of ${Z, Z}_{j}, under the condition ofM$. The inverse-variance weighted (IVW) estimator $\hat{\beta}_{IVW}$ as follows:

$\hat{\beta}_{IVW}=\frac{\sum_{j=1}^{J} \hat{\omega}_{j}\hat{\beta}_{j}}{\sum_{j=1}^{J} \hat{\omega}_{j}}, \hat{\beta}_{j}=\frac{\hat{\beta}_{Y_{j}}}{\hat{\beta}_{G_{j}}}, \hat{\omega}_{j}=\frac{\hat{\beta}_{G_{j}}^{2}}{\hat{\sigma}_{\hat{\beta}_{Y_{j}}}^{2}}$.

The IVW estimator is consistent under the no measurement error assumption, and we estimated $\beta_{G}=\sigma_{G}\beta$ based on the IVW estimator. Finally, we obtained the desired effect $\beta_{L}$by dividing the reasonable value of the genetic variance of the latent exposure $\theta$.

**2.4 Polygenic risk score calculation with k-folds cross-validation**

The corresponding PRS for individual *i* from testing samples, PRSi, can be characterized by:

$${PRS}_{i}=\sum_{j=1}^{J} w_{j}^{train}\times{SNP}_{i,j}$$

, which is similar to the original formula of the PRS; $w_{j}^{train}$ is the estimated coefficient of univariate logistic regression of binary exposure on the *j*th SNP, using the training samples, while SNPi, j is the genotype of testing sample *i* at the *j*th SNP.

**Supplementary Tables**

**Table 1. Detailed information on SNPs**

**Table 1.1 SNPs associated with gout**

| Exposure | SNP | Chr | Gene | Adjusted Covariates | Odds Ratio | P-value | Phenoscanner V2 Trait |
| --- | --- | --- | --- | --- | --- | --- | --- |
| Gout | rs3775948 | 4 | SLC2A9 | None | 0.7565 | 6.574*10^-26^ | Red blood cell count, Gout, Serum urate, Uric acid, Uric acid females in serum, Uric acid in serum, Renal function related traits urea, Serum uric acid levels, Uric acid levels, Self-reported gout, Treatment with allopurinol |
|  |  |  |  | Age, Sex | 0.7395 | 3.735*10^-28^ |  |
|  |  |  |  | Age, Sex, BMI | 0.7373 | 5.259*10^-28^ |  |
|  | rs2231142 | 4 | ABCG2 | None | 1.8103 | 5.273*10^-119^ | Gout, Gout male, Serum urate, Urate, Uric acid, Uric acid females, Uric acid females in serum, Uric acid in serum, Uric acid males, Uric acid males in serum, Serum uric acid levels, Serum uric acid levels in response to allopurinol in gout, Urate levels, Urate levels in lean individuals, Urate levels in obese individuals, Urate levels in overweight individuals, Uric acid levels, Basal metabolic rate, Body mass index, Impedance of leg left, Impedance of leg right, Impedance of whole body, Leg fat-free mass left, Leg fat-free mass right, Leg predicted mass left, Leg predicted mass right, self-reported gout, Treatment with allopurinol, Treatment with colchicine, Weight, Whole body fat-free mass, whole body water mass |
|  |  |  |  | Age, Sex | 1.9111 | 6.96*10^-129^ |  |
|  |  |  |  | Age, Sex, BMI | 1.9578 | 1.749*10^-134^ |  |
| Exposure | SNP | Chr | Gene | Adjusted Covariates | Odds Ratio | P-value | Phenoscanner V2 Trait |
| Gout | rs1165209 | 6 | SLC17A1 | None | 0.7883 | 3.321*10^-8^ | Hemoglobin concentration, Mean corpuscular hemoglobin, Mean corpuscular hemoglobin concentration, Mean corpuscular volume, Red cell distribution width, Height, Hip circumference adjusted for BMI, Hemoglobin Hb, Serum urate, Uric acid, Primary sclerosing cholangitis, Urate levels in lean individuals, Urate levels in overweight individuals, Arm fat-free mass left, Arm fat-free mass right, Arm predicted mass left, Arm predicted mass right, Basal metabolic rate, Disorders of mineral metabolism, Self-reported gout, Self-reported hereditary or genetic haematological disorder, Self-reported malabsorption or coeliac disease, Treatment with allopurinol, Trunk fat-free mass, Trunk predicted mass, Whole body fat-free mass, Whole body water mass |
|  |  |  |  | Age, Sex | 0.7743 | 7.338*10^-9^ |  |
|  |  |  |  | Age, Sex, BMI | 0.7697 | 4.848*10^-9^ |  |
|  | rs75786299 | 11 | SLC22A12 | None | 2.9037 | 1.771*10^-46^ | Not found |
|  |  |  |  | Age, Sex | 3.1899 | 2.583*10^-46^ |  |
|  |  |  |  | Age, Sex, BMI | 3.2838 | 7.272*10^-47^ |  |

**Table 1.2 Heterogeneous SNPs associated with gout**

| Exposure | SNP | Chr | Gene | Adjusted Covariates | Odds Ratio | P-value | Phenoscanner V2 Trait |
| --- | --- | --- | --- | --- | --- | --- | --- |
| Gout | rs671 | 12 | ALDH2 | None | 0.8503 | 2.602*10^-8^ | Alanine aminotransferase, Alanine aminotransferase Weekly alcohol intake 1484, Alanine aminotransferase alcohol drinkers, Alcohol, Alcohol consumption, Alcohol consumption female, Alcohol consumption male, Alcohol ever vs never, Aspartate aminotransferase, Coronary artery disease, Diastolic blood pressure, Drinking behavior, Esophageal squamous cell carcinoma Esophageal cancer, Gamma glutamyl transferase, Gamma glutamyl transferase drinkers, HDL cholesterol, HDL cholesterol adjusted for alcohol intake, HDL cholesterol drinkers, Intracranial aneurysm, LDL cholesterol, LDL cholesterol alcohol drinkers, Mean corpuscular hemoglobin concentration, Myocardial infarction, Serum creatinine, Serum creatinine estimated glomerular filtration rate eGFR, Serum triglycerides levels male drinkers, Serum triglycerides levels male drinkers 010 g of alcohol consumed per day, Serum triglycerides levels males, Systolic blood pressure, Uric acid in serum, Waist hip ratio, |
|  |  |  |  | Age, Sex | 0.8322 | 1.018*10^-9^ |  |

| Exposure | SNP | Chr | Gene | Adjusted Covariates | Odds Ratio | P-value | Phenoscanner V2 Trait |
| --- | --- | --- | --- | --- | --- | --- | --- |
| Gout | rs671 | 12 | ALDH2 | Age, Sex, BMI | 0.8416 | 1.353*10^-8^ | Alcohol consumption drinkers vs non drinkers, Alcohol consumption maxi drinks, Alcohol dependence, Body mass index, Coronary heart disease, Esophageal cancer, Hematological and biochemical traits, Mean corpuscular hemoglobin, Mean corpuscular volume, Metabolic syndrome, Renal function related traits sCR, Response to alcohol consumption flushing response, Serum alpha1 antitrypsin levels, Triglycerides, Esophageal neoplasms |

**Table 1.3 SNPs associated with HTN**

| Exposure | SNP | Chr | Gene | Adjusted Covariates | Odds Ratio | P-value | Phenoscanner V2 Trait |
| --- | --- | --- | --- | --- | --- | --- | --- |
| HTN | rs880315 | 1 | CASZ1 | None | 0.9177 | 4.152*10^-9^ | Diastolic blood pressure, Hypertension, Systolic blood pressure, log Urinary albumin creatinine ratio, Blood pressure, Pulse pressure, Urinary albumin to creatinine ratio, Illnesses of siblings: high blood pressure, Impedance of arm left, Impedance of leg left, Impedance of leg right, Impedance of whole body, Medication for cholesterol, blood pressure or diabetes: blood pressure medication, Medication for cholesterol, blood pressure or diabetes: none of the above, No treatment with medication for cholesterol, blood pressure, diabetes, or take exogenous hormones, Number of treatments or medications taken, Self-reported hypertension, Treatment with amlodipine, Treatment with atenolol, Treatment with bendroflumethiazide, Treatment with blood pressure medication, Treatment with doxazosin, Treatment with lisinopril, |
|  |  |  |  | Age, Sex | 0.9110 | 1.401*10^-9^ |  |
| Exposure | SNP | Chr | Gene | Adjusted Covariates | Odds Ratio | P-value | Phenoscanner V2 Trait |
| HTN | rs880315 | 1 | CASZ1 | Age, Sex, BMI | 0.9053 | 4.356*10^-10^ | Vascular or heart problems diagnosed by doctor: high blood pressure, Vascular or heart problems diagnosed by doctor: none of the above, Coronary artery disease |
|  | rs12037987 | 1 | WNT2B | None | 1.1122 | 1.090*10^-12^ | Cause of death: malignant neoplasm of thyroid gland, Diastolic blood pressure, Height, Illnesses of mother: high blood pressure, Illnesses of siblings: high blood pressure, Medication for cholesterol, blood pressure or diabetes: blood pressure medication, Medication for cholesterol, blood pressure or diabetes: none of the above, No treatment with medication for cholesterol, blood pressure, diabetes, or take exogenous hormones, Self-reported hypertension, Systolic blood pressure, Treatment with amlodipine, Treatment with bendroflumethiazide, Treatment with blood pressure medication, Vascular or heart problems diagnosed by doctor: high blood pressure, Vascular or heart problems diagnosed by doctor: none of the above |
|  |  |  |  | Age, Sex | 1.1237 | 1.628*10^-13^ |  |
|  |  |  |  | Age, Sex, BMI | 1.1392 | 1.955*10^-15^ |  |
| Exposure | SNP | Chr | Gene | Adjusted Covariates | Odds Ratio | P-value | Phenoscanner V2 Trait |
| HTN | rs11126666 | 2 | KCNK3 | None | 0.8786 | 6.684*10^-17^ | Body mass index in males, Body mass index, Kawasaki disease with coronary artery lesions, Diastolic blood pressure, Illnesses of father: high blood pressure, Illnesses of mother: high blood pressure, Illnesses of mother: none of the above, group 1, Illnesses of siblings: high blood pressure, Medication for cholesterol, blood pressure or diabetes: blood pressure medication, Medication for cholesterol, blood pressure or diabetes: none of the above, Pulse rate, Self-reported hypertension, Systolic blood pressure, Treatment with amlodipine, Treatment with bendroflumethiazide, Treatment with blood pressure medication, Treatment with doxazosin, Vascular or heart problems diagnosed by doctor: high blood pressure, Vascular or heart problems diagnosed by doctor: none of the above |
|  |  |  |  | Age, Sex | 0.8718 | 4.123*10^-17^ |  |
|  |  |  |  | Age, Sex, BMI | 0.8954 | 3.244*10^-15^ |  |
| Exposure | SNP | Chr | Gene | Adjusted Covariates | Odds Ratio | P-value | Phenoscanner V2 Trait |
| HTN | rs1902859 | 4 | RP11-576N17.4 | None | 1.1474 | 2.026*10^-22^ | Diastolic blood pressure, Systolic blood pressure, Hypertension, Illnesses of father: high blood pressure, Illnesses of mother: high blood pressure, Illnesses of siblings: high blood pressure, Illnesses of siblings: none of the above, group 1, Medication for cholesterol, blood pressure or diabetes: blood pressure medication, Medication for cholesterol, blood pressure or diabetes: none of the above, No treatment with medication for cholesterol, blood pressure, diabetes, or take exogenous hormones, Self- reported hypertension, Treatment with atenolol, Treatment with bendroflumethiazide, Treatment with blood pressure medication, Treatment with candesartan cilexetil, Treatment with ramipril, Vascular or heart problems diagnosed by doctor: high blood pressure, Vascular or heart problems diagnosed by doctor: none of the above, Coronary artery disease |
|  |  |  |  | Age, Sex | 1.1668 | 4.25*10^-25^ |  |
|  |  |  |  | Age, Sex, BMI | 1.1680 | 9.063*10^-24^ |  |
| Exposure | SNP | Chr | Gene | Adjusted Covariates | Odds Ratio | P-value | Phenoscanner V2 Trait |
| HTN | rs1361831 | 6 | RP11-394G3.2 | None | 1.0878 | 1.764*10^-9^ | Hemoglobin concentration, Waist hip ratio in females, Waist hip ratio, Waist hip ratio adjusted for BMI, Diastolic blood pressure, Heel bone mineral density, Height, Illnesses of mother: high blood pressure, Illnesses of mother: none of the above, group 1, Illnesses of siblings: high blood pressure, Illnesses of siblings: none of the above, group 1, Medication for cholesterol, blood pressure or diabetes: blood pressure medication, No treatment with medication for cholesterol, blood pressure, diabetes, or take exogenous hormones, Self-reported hypertension, Systolic blood pressure, Treatment with amlodipine, Treatment with atenolol, Treatment with bendroflumethiazide, Treatment with blood pressure medication, Vascular or heart problems diagnosed by doctor: high blood pressure, Vascular or heart problems diagnosed by doctor: none of the above |
|  |  |  |  | Age, Sex | 1.0838 | 1.263*10^-8^ |  |
|  |  |  |  | Age, Sex, BMI | 1.0832 | 3.860*10^-8^ |  |
| Exposure | SNP | Chr | Gene | Adjusted Covariates | Odds Ratio | P-value | Phenoscanner V2 Trait |
| HTN | rs3740392 | 10 | AS3MT | None | 1.0957 | 1.260*10^-9^ | High light scatter percentage of red cells, High light scatter reticulocyte count, Immature fraction of reticulocytes, Mean corpuscular hemoglobin, Mean corpuscular volume, Reticulocyte count, Reticulocyte fraction of red cells, Ever smoked, Maternal smoking around birth, Past tobacco smoking, Smoking status: previous, Tobacco smoking: ex-smoker |
|  |  |  |  | Age, Sex | 1.1046 | 4.236*10^-10^ |  |
|  |  |  |  | Age, Sex, BMI | 1.1237 | 1.535*10^-12^ |  |
|  | rs6585255 | 10 | NHLRC2 | None | 1.1187 | 1.13*10^-8^ | Not found |
|  |  |  |  | Age, Sex | 1.140 | 2.703*10^-10^ |  |
|  |  |  |  | Age, Sex, BMI | 1.1511 | 6.373*10^-11^ |  |
|  | rs4980389 | 11 | LSP1 | None | 0.9090 | 1.378*10^-9^ | Diastolic blood pressure, Illnesses of mother: breast cancer, Illnesses of mother: high blood pressure, Illnesses of siblings: high blood pressure, Impedance of arm left, Impedance of arm right, Medication for cholesterol, blood pressure or diabetes: blood pressure medication, No treatment with medication for cholesterol, blood pressure, diabetes, or take exogenous hormones, Self-reported hypertension, Systolic blood pressure, Treatment with amlodipine, Treatment with bendroflumethiazide, Treatment with blood pressure medication, Treatment with doxazosin, Treatment with perindopril, |
|  |  |  |  | Age, Sex | 0.8995 | 1.846*10^-10^ |  |
| Exposure | SNP | Chr | Gene | Adjusted Covariates | Odds Ratio | P-value | Phenoscanner V2 Trait |
| HTN | rs4980389 | 11 | LSP1 | Age, Sex, BMI | 0.8907 | 1.72*10^-11^ | Vascular or heart problems diagnosed by doctor: high blood pressure, Vascular or heart problems diagnosed by doctor: none of the above |
|  | rs17249754 | 12 | ATP2B1 | None | 0.9008 | 7.31*10^-12^ | High light scatter percentage of red cells, High light scatter reticulocyte count, Reticulocyte count, Reticulocyte fraction of red cells, Diastolic blood pressure, Hypertension, Pulse pressure, Systolic blood pressure, Coronary artery disease, Myocardial infarction, Mean arterial pressure, Systolic blood pressure diastolic blood pressure and hypertension,  Biomedical quantitative traits, Blood pressure, Illnesses of siblings: high blood pressure, Medication for cholesterol, blood pressure or diabetes: blood pressure medication, Medication for cholesterol, blood pressure or diabetes: none of the above, Self-reported hypertension, Treatment with bendroflumethiazide, Treatment with blood pressure medication, Treatment with doxazosin, Treatment with perindopril, Vascular or heart problems diagnosed by doctor: high blood pressure, Vascular or heart problems diagnosed by doctor: none of the above |
|  |  |  |  | Age, Sex | 0.8867 | 6.19*10^-14^ |  |
|  |  |  |  | Age, Sex, BMI | 0.8893 | 1.622*10^-12^ |  |
| Exposure | SNP | Chr | Gene | Adjusted Covariates | Odds Ratio | P-value | Phenoscanner V2 Trait |
| HTN | rs35427 | 12 | RP11-25E2.1 | None | 0.8976 | 4.983*10^-11^ | Platelet count, Platelet crit, Diastolic blood pressure, Illnesses of mother: high blood pressure, Impedance of leg left, Impedance of leg right, Impedance of whole body, Medication for cholesterol, blood pressure or diabetes: blood pressure medication, Medication for cholesterol, blood pressure or diabetes: none of the above, No treatment with medication for cholesterol,  blood pressure, diabetes, or take exogenous hormones, Number of treatments or medications taken, Self-reported hypertension, Systolic blood pressure, Treatment with amlodipine, Treatment with bendroflumethiazide, Treatment with blood pressure medication, Treatment with doxazosin, Treatment with lisinopril, Vascular or heart problems diagnosed by doctor: high blood pressure, Vascular or heart problems diagnosed by doctor: none of the above |

**Table 2. P-values for the association between SNPs and measured confounders.**

| **Exposure** | **SNP** | **Age** | **BMI** | **Creatinine** | **Sex** |
| --- | --- | --- | --- | --- | --- |
| **Gout** | **rs3775948** | 0.8288 | 0.6066 | 0.0301 | 0.8288 |
|  | **rs2231142** | 0.2727 | 0.0499 | 0.2727 | 0.8288 |
|  | **rs1165209** | 0.3485 | 0.8288 | 0.4345 | 0.8836 |
|  | **rs75786299** | 0.3719 | 0.7439 | 0.3719 | 0.8288 |
|  | **rs671** | 0.8645 | 0.3485 | 0.0045 | 0.7116 |
| **Hypertension** | **rs880315** | 0.8827 | 0.8827 | 0.9415 | 0.9674 |
|  | **rs12037987** | 0.9511 | 0.8865 | 0.9568 | 0.9511 |
|  | **rs11126666** | 0.9090 | 0.1777 | 0.8299 | 0.8299 |
|  | **rs1902859** | 0.9511 | 0.8827 | 0.0920 | 0.9674 |
|  | **rs1361831** | 0.0488 | 0.7894 | 0.4766 | 0.9442 |
|  | **rs3740392** | 0.9511 | 0.3097 | 0.1064 | 0.9827 |
|  | **rs6585255** | 0.8299 | 0.9511 | 0.9511 | 0.9827 |
|  | **rs4980389** | 0.9827 | 0.4241 | 0.8299 | 0.9511 |
|  | **rs17249754** | 0.7894 | 0.1448 | 0.9511 | 0.9511 |
|  | **rs35427** | 0.9568 | 0.4716 | 0.9442 | 0.9511 |

**Table 3. Comparing no remove and remove SNPs which associated with exposure correlated with measured confounders**

| **Exposure** | **Gout** | | **HTN** | |
| --- | --- | --- | --- | --- |
| **Condition** | **no remove** | **remove** | **no remove** | **remove** |
| **SNPs** | rs3775948 | rs3775948 | rs880315 | rs880315 |
|  | rs2231142 | rs2231142 | rs12037987 | rs12037987 |
|  | rs1165209 | rs1165209 | rs11126666 | rs11126666 |
|  | rs75786299 | rs75786299 | rs1902859 | rs1902859 |
|  | rs671 |  | rs1361831 | rs1361831 |
|  |  |  | rs3740392 | rs3740392 |
|  |  |  | rs6585255  rs4980389 | rs6585255  rs4980389 |
|  |  |  | rs17249754 | rs17249754 |
|  |  |  | rs35427 | rs35427 |

**Supplementary Figures**

MR analysis with coarsened exposures from hypertension to gout without measured confounders after removing SNPs associated with measured confounders.88,347 participants and 686,439 SNPs sampled from the Taiwan Biobank

SNP exclusion criteria:

Genotype missing rate > 0.1: 12674

Hardy–Weinberg equilibrium < 10^-6^: 18731

Minor allele frequency < 0.01: 178422

88,347 participants and 476612 SNPs eligible for research

Without adjusted covariates

Adjust sex and age

Adjust sex, age, and BMI

SNP inclusion criteria:

p-value < 5*10^-8^

Weak linkage disequilibrium

(R^2^ < 0.001)

SNP inclusion criteria:

p-value < 5*10^-8^

Weak linkage disequilibrium

(R^2^ < 0.001)

SNP inclusion criteria:

p-value < 5*10^-8^

Weak linkage disequilibrium

(R^2^ < 0.001)

five SNPs associated with gout;

ten SNPs associated with hypertension

**Figure 1**. Flow chart for GWAS procedure of SNP selection under three different settings: unadjusted, adjusted to sex and age, and adjusted to sex, age, and BMI.


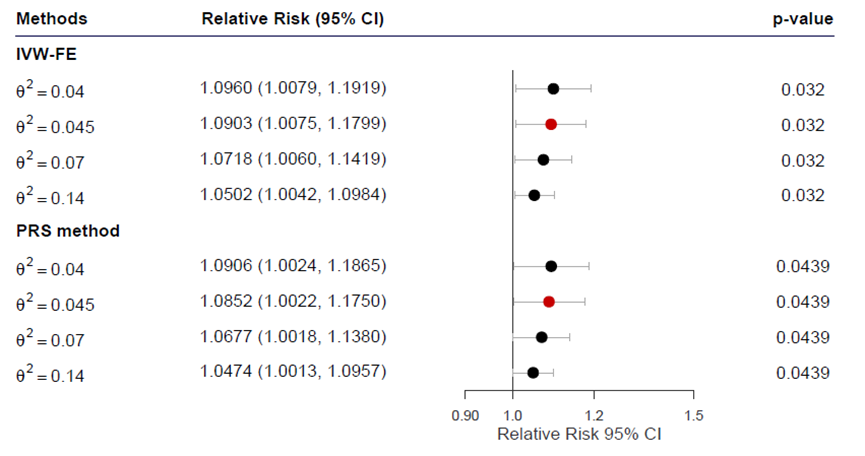


**Figure 2**. MR analysis with coarsened exposures from gout to hypertension without measured confounders after removing SNPs associated with measured confounders.

IVW-FE, IVW fixed-effects method; PRS, polygenic risk scores; CI, confidence interval.

**References**

1. Tudball MJ, Bowden J, Hughes RA, Ly A, Munafo MR, Tilling K et al: Mendelian randomisation with coarsened exposures. Genet Epidemiol 2021, 45:338-50.

2. Lee SH, Goddard ME, Wray NR, Visscher PM: A better coefficient of determination for genetic profile analysis. Genet Epidemiol 2012, 36:214-24.
